# Behavior Under Repeated Loading of RC Beams Externally Strengthened in Flexure with SRG Systems

**DOI:** 10.3390/ma16041510

**Published:** 2023-02-10

**Authors:** Francesco Bencardino, Mattia Nisticò

**Affiliations:** Department of Civil Engineering, University of Calabria, Via P. Bucci, Cubo 39B, 87036 Rende, Cosenza, Italy

**Keywords:** repeated loading, external strengthening, steel-reinforced grout

## Abstract

Steel-reinforced grout (SRG) systems are effective methods for the flexural strengthening of reinforced concrete (RC) beams. In this study, the effect of a limited number of repeated loads on the structural response and debonding evolution of strengthened beams was experimentally investigated. The number of available research concerning the cyclic behavior of SRG-strengthened members is quite limited and this research attempts to cover this knowledge. A total of ten full-scale RC beam specimens were tested under a four-point bending scheme. The effectiveness of the traditional externally bonded (EB) strengthening technique was compared with a promising innovative technique referred to as inhibiting/repairing/strengthening (IRS). The test variables included the use of two SRG configurations using high and low steel strip density. The experimental results revealed that the performance of the beams was largely dependent on the spacing of the steel strands within the reinforcing strip. Under repeated loading, the debonding of the external system takes place when steel fiber with high mass per unit of area was used. By increasing the matrix impregnation of the steel strip, the composite system was not affected by debonding. Further, the efficiency in terms of flexure enhancement, local and global ductility performance and energy dissipation of the beams are also discussed.

## 1. Introduction

Most of the structures built in the last century are the result of the use of reinforced concrete (RC) as construction material. These structures, including buildings, bridges, water tanks, etc. have been made with huge investments of economic and material resources. Among them, bridges and viaducts represent a significant part of the total infrastructure.

The span between two piers is covered by beams or girders that represent the main components of the superstructure. Due to their external exposure, girder members are usually vulnerable to actions such as freeze and thaw shrinkage, elevated temperature, humidity, salt and acid rain. The performance decay associated with exposure to aggressive environments represents an important issue extensively studied in recent years [1,2,3,4,5]. The principal goal of this research is to develop durability test procedures and protocols in the current code guidelines. Additionally, these structures are significantly affected by nonconventional loading such as cyclic actions. They are represented by the moving load on the bridge throughout its length. The environmental actions and service loads could severely damage the structural members, which need to be repaired by effective strengthening interventions.

Starting from traditional interventions such as steel plate bonding, the use of continuous long fibers applied by means of polymeric or cementitious matrices has increased rapidly as a common technique to strengthen structural members. In this context, externally applied innovative materials were born as a local reinforcement for structures not lacking in stiffness such as bridge decks. Subsequently, their application has gained widespread attention also in the retrofitting of buildings. Typically, both types of structures are also particularly vulnerable to seismic load due to inadequate flexural reinforcement.

In the last years, several pieces of research have directed efforts on systems composed of cementitious matrices coupled with fibers of various properties, commonly recognized by the acronym fabric-reinforced cementitious matrix (FRCM) or textile-reinforced mortar (TRM). The high tensile strength of the FRCM/TRM composites enables the existing civil infrastructure to carry the currently designed loads. Usually, when the fiber reinforcement is made of steel strands (stainless steel or ultrahigh tensile strength steel), the system is also called steel-reinforced grout (SRG). From here forward, the acronym SRG is used.

The effectiveness of cementitious-based composite strengthening systems for the upgrading of the flexural capacity of RC beams found large interest in the scientific community. These studies confirmed the goodness of these applications through several publications [6,7,8]. The experimental evidence was investigated considering the wide range of materials available on the market (fibers and cementitious matrices). 

Therefore, the experimental investigation of RC beams under in-plane repeated or cyclic loads is relevant to better study and understand their global and local behavior. The variability of the applied loads can lead to damage to the external reinforcements. The knowledge base starts with the study of the fatigue behavior of RC beams [9,10]. As reported in [11], this study highlighted that failure is a result of a fatigue fracture in the tension steel reinforcement with a sudden failure of the section once the fracture has spread in the bars. In particular, the fatigue performance of strengthened RC beams with externally bonded fiber-reinforced polymer (FRP) systems was addressed in [12,13,14,15]. Specifically, these studies confirmed that the presence of the external FRP material increases the fatigue life of the beams with similar failure modes of the unstrengthened beams, reducing the stresses on the steel reinforcing bars. In other works, the experimental results lead to observations of FRP delamination before the fatigue failure of the steel reinforcement [16]. Many more studies have evaluated the fatigue behavior of the FRP/FRCM beam-column joint and masonry specimens [17,18,19,20,21]. An interesting study of the fatigue behavior of RC beams strengthened in flexure with steel-reinforced inorganic polymers (SRPs) [6] concluded and confirmed that the beams failed due to fatigue of the steel reinforcement with a partial debonding of the external fibers contributing to extending the strength of the beams and extending their fatigue life. Among this range of composite systems, unidirectional steel fibers coupled with inorganic matrices have gained widespread attention in the scientific literature due to many advantages such as better vapor permeability of the substrate with respect to that obtained with SRPs, application on wet substrates, resistance to high temperature [22] and UV exposure, greater reversibility of the application and improvement of the composite durability in aggressive environments thanks to a high final thickness [23,24].

In general, many studies and pieces of research have confirmed the structural effectiveness of both RC beams and columns [25,26,27,28]. 

From this knowledge, the authors believe it is necessary to start an experimental campaign considering the limited studies of RC beams subjected to repeated flexural loading strengthened with external SRG systems. Moreover, no studies have treated the SRG issue. The repetition of the load on the structures could cause significant damage to the SRG strengthening system. As widely known, the effectiveness of external bonding strengthening systems is strictly influenced by the bond condition and severely dependent on the fiber/matrix interface quality.

According to the best of the authors’ knowledge, this investigation is the first attempt to understand the structural response of strengthened RC beams by means of SRG systems subjected to a limited number of repeated loads. The experiment was conducted to better highlight the influence of the strengthening technique and geometrical properties of the steel fiber on the debonding phenomena, strength, ductility and stiffness of the beams. To this aim, a preliminary study of the structural performance of RC beams subjected to a limited number of load cycles was carried out. The tests included two groups (A and B) of full-scale beams strengthened in flexure. The external strengthening systems were applied according to the traditional externally bonded (EB) and an innovative inhibiting/repairing/strengthening (IRS) technique. The investigated strengthening techniques are described in the paper according to the different executive phases. 

The IRS technique can be associated with a near-surface-mounted (NSM) technique by using a matrix that contains corrosion inhibitors for the steel reinforcement bars. It allows the realization of a promising strengthening technique both from a structural and environmental point of view. These properties make it particularly suitable in interventions in which the restoration of the cover concrete is required.

The external reinforcement used in this study involves two steel tapes with high and low strip density. The aim is to assess the effectiveness of SRG strengthening systems under repeated loading, the influence of premature delamination and the failure modes involved. The results were finally compared with two control beams numerically simulated under monotonic loading and two monotonically tested beams strengthened by means of the EB procedure.

## 2. Experimental Program

The experimental activities aimed to analyze the behavior of full-scale RC beams strengthened in flexure with SRG systems. The geometrical dimensions of the beams and external reinforcement ratio were designed to investigate a potential scale effect in terms of flexural moment capacity and ductility, keeping the same internal reinforcing steel bars (in compression and tension). Ten beams were realized and strengthened with two different steel strips (hereafter labeled SS and GLV) using the classic externally bonded technique indicated as EB and an innovative IRS technique similar to the NSM technique. The aim is to investigate the structural performance and effectiveness of EB-SRG and IRS-SRG systems of two full-scale RC beam groups. The tests were conducted under four-point monotonic and repeated loading. The experimental procedures were planned and carried out at the laboratory of “Testing Materials and Structures” of the University of Calabria—UniCal, Italy. More details are described in the paragraphs below.

### 2.1. Geometries and Schedule of Tests

**Figure 1:**
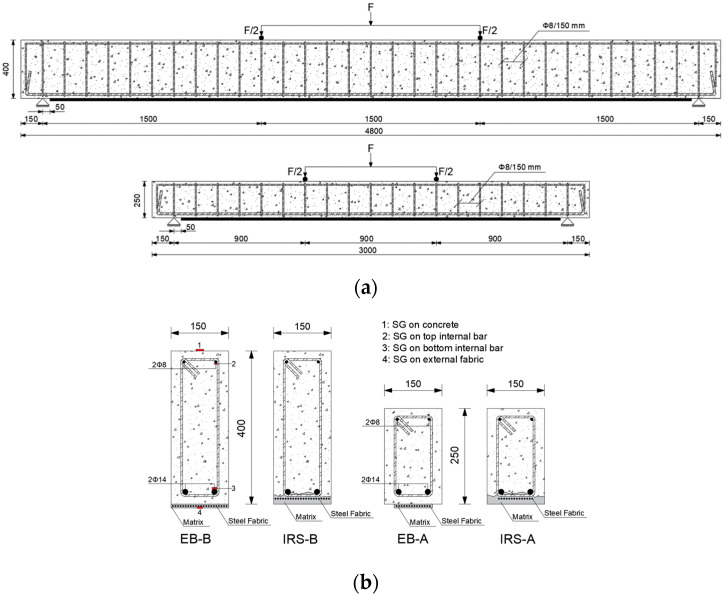
(**a**) Beam geometry and steel reinforcement layout and (**b**) cross-section of the beams, strengthening techniques and SGs’ position.

This study comprises ten flexural tests of RC beams divided into two groups (A and B). The beams of group A were 3000 mm in length (L), 250 mm in height (h) and 150 mm in width (b), whilst those in group B were designed with dimensions of 4800 mm length (L), 400 mm height (h) and 150 mm width (b) (overall dimensions are sketched on Figure 1 and summarized in Table 1). The clear span (l) was 2700 mm and 4500 mm for groups A and B, respectively. The shear spans (a), over which the load was applied, were 900 mm and 1500 mm, respectively. All beams were internally reinforced with two longitudinal bars in tension (diameter of 14 mm) and two longitudinal bars in compression (diameter of 8 mm) of steel grade “B450C”. The beams were designed to fail in flexure before in shear using a four-point load configuration. The shear reinforcement consisted of 8 mm steel stirrups arranged at 90 degrees and placed at intervals of 150 mm. 

The beams were strengthened on the tension side by applying steel strips of 100 mm and 150 mm width (b_f_) for groups A and B, respectively. The external strengthening was 2600 mm and 4400 mm long (l_f_), respectively, shorter than the clear span, in order to reproduce the real arrangement in the bridge decks and to avoid contact with the steel supports during the rotation of the beams. The SRG systems were applied to the strengthened beams after the complete aging of the concrete. Figure 1 also shows the layout of the external reinforcement. All beams were strengthened using one ply of steel strip. 

The effective depth (d) and the external reinforcing area (A_f_) of both groups were changed to consider the differences in effective strengthening capacity and keep constant the external strengthening ratio (ρ_f_ = b_f_ × t_f_/b × d) and the shear span length to effective depth (a/d) ratio. This geometric and reinforcement configuration of the two groups of beams was chosen to identify a possible scale effect and to compare the performance and the results of all beam specimens.

The beams tested under repeated loading were labeled according to the following structure: X_Y_A/B. ‘X’ represents the strengthening technique used (EB for “Externally Bonded” and IRS for “Inhibiting/Repairing/Strengthening”), while ‘Y’ refers to the steel strip used as external reinforcement (SS for “stainless steel” and GLV for “galvanized steel”). Finally, A and B indicate the group to which the beam belongs. The acronyms preceded by the letter ‘M’ indicate the two beams tested with monotonic loading. 

To summarize, the set of ten specimens includes eight strengthened beams tested under repeated load, of which four are tested with the traditional EB technique (EB_SS_A, EB_SS_B, EB_GLV_A and EB_GLV_B) and four are tested with the novel IRS technique (IRS_SS_A, IRS_SS_B, IRS_GLV_A and IRS_GLV_B), and two strengthened beams tested under monotonic load with the EB technique and stainless steel strip (M-EB_SS_A and M-EB_SS_B). The test program was arranged in order to carry out experimental comparisons between the two groups of beams (A and B), the two reinforcement techniques (EB and IRS) and the types of reinforcement fibers (stainless and galvanized). To complete the experimental schedule, two unstrengthened benchmark beams (hereafter labeled as CB_A and CB_B) were analyzed using nonlinear finite element analysis. The control beams and the monotonically tested beams were used for the assessment of the structural performance of the other strengthened beams.

### 2.2. Concrete and Internal Steel Bars’ Properties

Samples of concrete and internal steel bars were experimentally tested in order to evaluate the mechanical properties. The compressive (f_cm_) and tensile (f_ctm_) strength of the concrete were evaluated from 12 cylinders with a dimension of 150 mm × 300 mm (diameter × height), cured under the same laboratory conditions as the beams and sourced from the same concrete batch. Specifically, the tensile strength was determined with indirect splitting tests. The specimens were tested at 28 days and on the day of testing, as summarized in Table 2. The specimens of internal steel bars were dried in an oven and weighed. Tension tests were conducted for both diameters (Φ8 and Φ14) in order to evaluate the average yield (f_ym_) and ultimate (f_tm_) tensile strength on six samples (three for each diameter), as listed in Table 2. In round brackets, the coefficients of variations (C.o.V.) are reported.

### 2.3. Steel-Reinforced Grout (SRG) Properties

**Figure 2:**
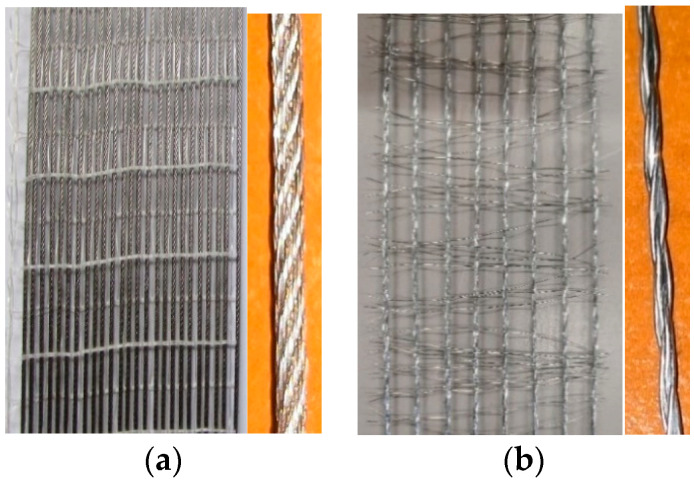
Steel textile and details of the single strand: (**a**) SS and (**b**) GLV.

Two types of steel strips (Figure 2), differing in their mechanical and geometrical properties, were used as external reinforcement for the beams. Both are unidirectional fibers made of steel strands embedded in the same cementitious inorganic matrix. All materials were supplied by Kimia S.p.A. The first reinforcement is made of stainless steel (SS) strands, particularly resistant to corrosion, which can be used in interventions on surfaces subject to rising damp and, in general, exposed to aggressive environments. Each strand is composed of seven wires and, in turn, made up of seven other threads twisted around each other. The tensile strength (f_f_), elastic modulus (E_f_) and rupture strain (ε_f_) are equal to 1430.53 MPa (C.o.V. = 0.074), 206794 MPa (C.o.V. = 0.059) and 0.0148 (C.o.V. = 0.088), respectively. On the other hand, the second steel reinforcement consists of five high-strength galvanized zinc-plated (GLV) filaments rolled up longitudinally. GLV reinforcement has recently been released commercially by the manufacturer, so there are no other publications investigating its structural response. It is characterized by a tensile strength of 1988.06 MPa (C.o.V. = 0.036), elastic modulus of 198,352 MPa (C.o.V. = 0.025) and strain at failure of 0.0153 (C.o.V. = 0.035). The mechanical properties were obtained by testing 3 specimens for each steel fiber by means of direct tensile tests. Table 3 provides the measured properties as well as the equivalent thickness (t_f_), the mass per unit of area (ω_f_), the cord spacing (s) and the transversal area of a single strand (A_f,str_). Further details were provided by the manufacturer in the technical datasheets [29,30]. As reinforcement type, the two fibers can be classified as high-density fiber (SS) and low-density fiber (GLV), as observed from the strip density (ω_f_ in Table 3). The GLV reinforcement is selected to facilitate the matrix penetration and ensure better steel strip/surface adhesion after comparing the experimental results with the high-density SS fibers. 

In both cases, the steel strips were applied with a nonshrink, normal curing, ready-to-use, thixotropic matrix with the addition of synthetic fibers and mineral-natured inorganic polymers [31]. It has high mechanical strength for both short and long curing times, strong adhesion to concrete, high resistance against sulfates and excellent durability even in severe aggressive conditions (coastal areas, deicing salts and acid rain). The recommended matrix is made of fine particles (size range 0.1–0.5 mm). The matrix also acts as an inhibitor of corrosion and represents an ecofriendly material for green technology as demonstrated in several studies [32,33]. Therefore, it is suitable for operations of passivation of internal steel bars, restoration and repair, and at the same time, compatible with the IRS technique (see paragraph 2.4). The mechanical properties, compressive strength (f_cmm_) and flexural tensile strength (f_tmm_) were obtained by testing prismatic specimens with a dimension of 160 × 40 × 40 mm^3^ according to [34] (Table 4). Finally, the estimation of the elastic secant modulus (E_m_) in compression was performed on cubic specimens (150 mm × 150 mm × 150 mm) using linear strain gauges (SGs).

### 2.4. Surface Preparation and Strengthening Operations

**Figure 3:**
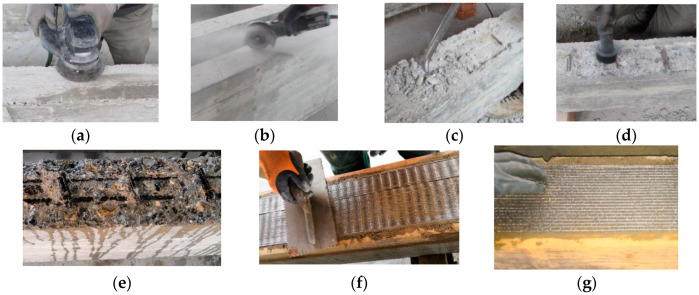
Preparation of the beams and installation of the SRG strengthening systems: (**a**) light mechanical scarification (EB); (**b**) carvings to improve bond (EB); (**c**) removing of the residual cover concrete (IRS); (**d**) removing of dust; (**e**) wetting with water; (**f**) application of SS strip; and (**g**) application of GLV strip.

The strengthening phases were performed with the tensile zone of the beams turned upside down in order to facilitate the work procedures and minimize errors in the application of the steel strip reinforcements. Indeed, the preparation of the surfaces and the technique of applying the composite material represent a fundamental phase of the strengthening process. Before the installation of the SRG systems, the beams were cleaned on the bottom side from any type of material (dust and other substances) and wet abundantly with water before the spreading of the cementitious matrix to avoid absorption of the mixing water and to ensure an effective application. All reinforcement textiles were bonded to the surfaces without anchors. The relevant steps related to the strengthening techniques are shown in Figure 3.

#### 2.4.1. Traditional Externally Bonded (EB) Technique

According to the traditional strengthening technique (EB), two layers (each one of about 5 mm) of the cementitious matrix are applied. The beams tested with the EB technique were subjected to light mechanical scarification and carvings in order to ensure optimum bond at the interface of concrete/composite systems. The strengthening phases can be summarized in the following points:

- Preparation of the external surface (Figure 3a,b).
- Installation of the external strengthening:
  (a) Application of the first layer (upper layer) of the matrix with a suitable average thickness (thickness of 5 mm).
  (b) Application of steel strip reinforcement over the first layer of the wet matrix, ensuring the full impregnation of the steel fiber by pressing with a metal trowel (Figure 3g).
  (c) Application of the second layer (bottom layer) of the inorganic matrix (thickness of 5 mm).

#### 2.4.2. Inhibiting-Repairing-Strengthening (IRS) Technique

A new innovative application technique (IRS) was experimentally tested with effective results [35] and reproposed in this study. It consists of the restoration of the deteriorated concrete and application of the external strengthening in one step with the installation of the steel strips in the cover concrete with a reduction in times and costs, number of used materials and the amount of chemical compounds. Using a suitable matrix (thixotropic with passivation properties), the operations of steel bar corrosion inhibition/protection can be performed at the same time as the restoration of the deteriorated concrete and installation of the external strengthening. The final layer of the matrix shows more consistent thicknesses (about 30–40 mm). With reference to the IRS-strengthened beams, the specimens were cast in the molds without the cover concrete. After that, the remaining concrete was removed up to the longitudinal and transverse steel reinforcement (Figure 3c), which is then successively covered by the matrix. The idea is to simulate the existing RC elements with damaged cover concrete, which, in the strengthening phase, is totally removed. The steps can be summarized as follows:

- Preparation of the external surface (Figure 3c,d).
- Installation of the external strengthening:
  (a) Application of the first layer (upper layer) of the inorganic matrix (thickness of 20–30 mm).
  (b) Application of steel strip reinforcement over the first layer of the wet matrix, ensuring the full impregnation of the steel fiber by pressing with a metal trowel (Figure 3f).
  (c) Application of the second layer (bottom layer) of the inorganic matrix (thickness of 5–10 mm).

### 2.5. Test Setup and Loading Procedure

**Figure 4:**
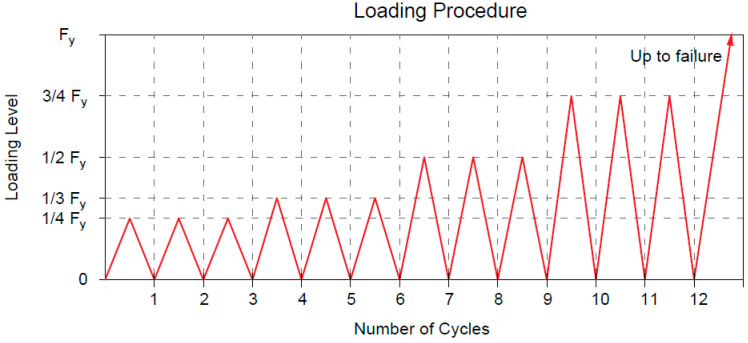
Loading setup of the specimens tested under repeated load.

Eight beams were loaded according to the setup shown in Figure 4 and their structural behavior was investigated. The action applied consists of repeated loads of increasing amplitude. Three cycles at the same amplitude are applied for each increment (step) in order to investigate the flexural strength. Specifically, the beams were tested according to 12 positive monotonic cycles. The periodic amplitudes were defined according to the theoretical yielding loads (F_sy_), using four levels of load corresponding to around 25% (F_sy_/4), 33.33% (F_sy_/3), 50% (F_sy_/2) and 75% (3F_sy_/4) of them. 

From the theoretical calculations, the yield loads of both groups of beams (A and B) were almost equal. The theoretical average yielding load was defined considering the self-weight of the RC beams and the self-weight of the steel beam used to distribute/subdivide the load. The self-weight was estimated as 25 kN/m^3^·b·h·L, obtaining 2.81 kN and 7.20 kN (group A and B, respectively). The steel beam was computed with weights of 0.27 kN and 0.80 kN, respectively. The values of the load cycles correspond to 12, 18, 30 and 42 kN for beams strengthened with stainless steel strips and 10, 16, 28 and 40 kN for beams strengthened with high-strength galvanized steel strips. The load history was repeated 3 times with 36 cycles in total, and later, the beams were monotonically loaded up to failure.

The repeated loading is chosen according to the provisions provided by Eurocode 2-1-1 [36] and ACI 549.4R-20 [37], which state that the stress levels in the internal steel reinforcement bars shall be limited to 80% of the yield strength under service condition.

**Figure 5:**
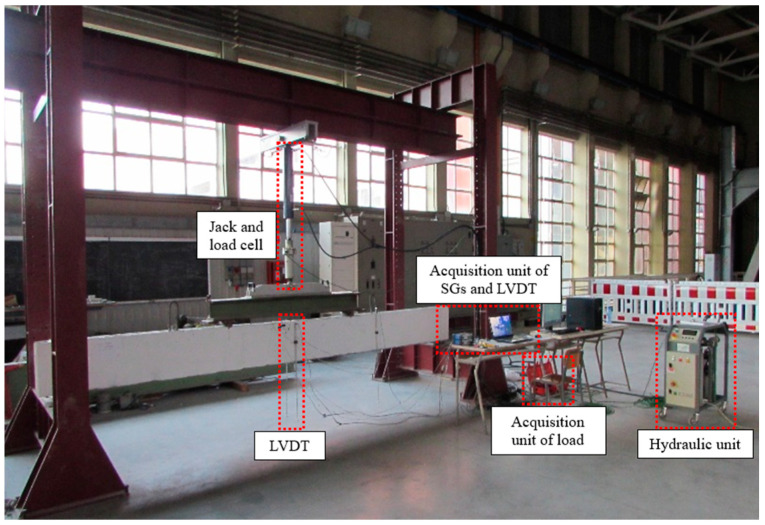
Experimental test setup.

The vertical load was gradually applied and released using a hydraulic unit that controls a 140 kN jack and 160 kN load cell connected to a suitable reaction steel frame for the application of the load on the beams, according to force-controlled loading. This equipment allows one to apply a load and keep it constant. The static scheme of four-point bending is performed by means of a stiff steel beam able to divide the applied load. The beams are simply supported at the ends using two steel cylinders. Figure 5 shows the test setup. 

The vertical displacements were recorded using 3 linear variable displacement transducers (LVDTs) of 500 mm capacity. Two were placed at midspan (both on the front and back side in order to identify unwanted rotations during the loading) and one at quarter span. Finally, the strain values of all materials (concrete, internal tension/compression steel reinforcement and external strengthening) were measured by means of electrical strain gauges arranged in the middle section of the beams, with lengths of 100 mm, 6 mm and 10 mm, respectively (Figure 1b). The data (loads, deflections and strains) are recorded with a frequency acquisition of 2 Hz by two acquisition systems: a control unit that manages strain gauges and LVDTs and a system for structural monitoring for the control and recording of the applied load (jack and load cell). In the specimens monotonically tested, the load was periodically paused in order to identify and mark the crack pattern, while in the repeated loading tests, it was performed at the end of each step, keeping the load at the cycle amplitude.

## 3. Test Results and Discussion

### 3.1. Structural Behavior

**Figure 6:**
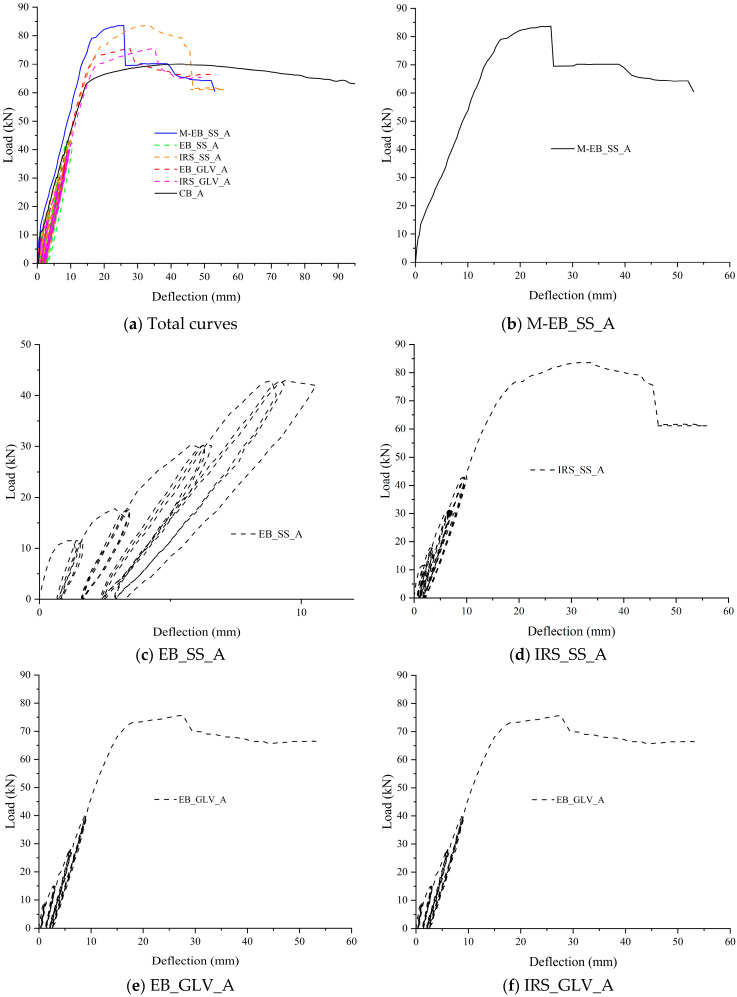
Load versus midspan deflection curves for group A specimens.

**Figure 7:**
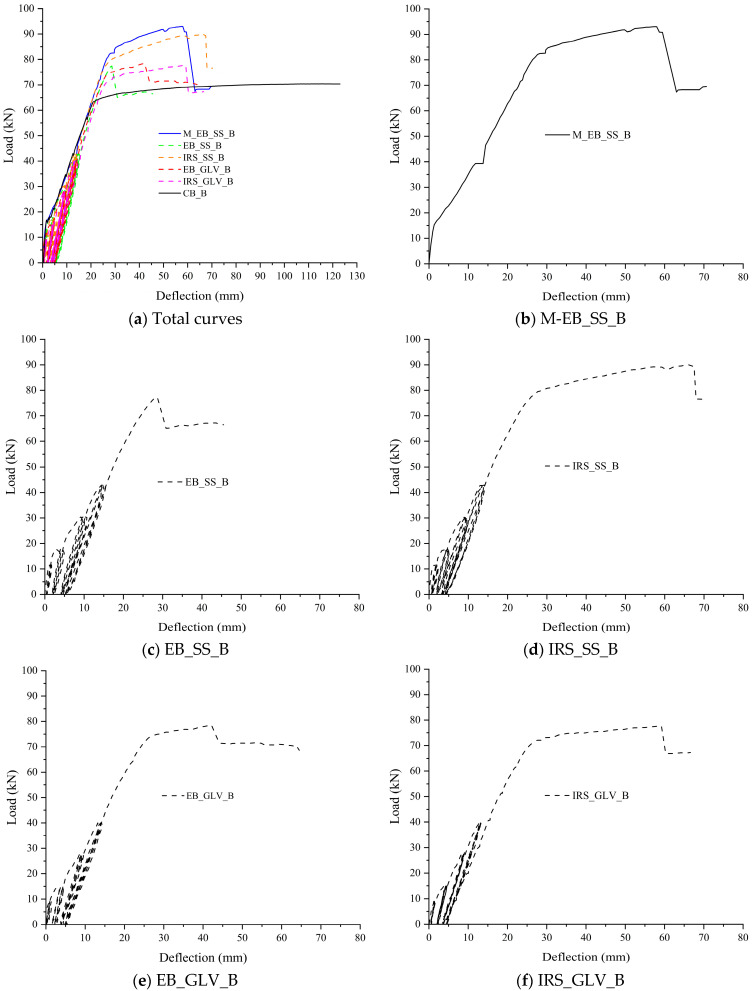
Load versus midspan deflection curves for group B specimens.

The experimental results obtained from the tested beams are critically analyzed and discussed in terms of load–deflection curves. Figure 6 and Figure 7 depict the applied load (recorded by the load cell as the total load) and the corresponding midspan deflection (as the average of the two LVDTs). The structural curves are grouped for both A and B specimens. The graphs show the response during the first 12 load cycles overlapped with the structural performance under monotonic loading up to failure. The further 24 load cycles are not added to have a better resolution of the curves.

The behavior of the strengthened beams can be compared with the control beams (CB_A and CB_B) and the EB-strengthened monotonic beams (M/EB_SS_A and M/EB_SS_B) in terms of yield load, ultimate load, respective deflections and global/local ductility.

Table 5 collects the values of loads and midspan deflections corresponding to the yielding of the steel bars in tension (F_sy_ and δ_sy_) and at failure (F_u_ and δ_u_). Further, the same table summarizes the deflection ductility ratio (μ_δ_ = δ_u_/δ_sy_), the ratio between the failure load of the strengthened beams and the corresponding control beams (Δ_F_ = F_u_/F_u,control_) and the ratio of the μ_δ_ of each strengthened beam versus the corresponding control beam (Δ_μ_ = μ_δ_/μ_δ,control_). Finally, the failure modes observed during tests are labeled in the last column of the table and discussed in paragraph 3.3. For the specimens tested monotonically, the load and midspan deflection at the first crack formation were also reported (F_cr_ and δ_cr_, respectively).

The yield load was identified graphically when a loss of stiffness occurred in correspondence with a change of slope in the load–deflection curve while the failure is accompanied/followed by a clear drop due to the sudden detachment or tensile rupture of the strengthening system.

As expected, the two monotonically tested beams (M/EB_SS_A and M/EB_SS_B) showed failure behavior governed by intermediate debonding of the external reinforcement. Bencardino and Condello [35] investigated the flexural behavior of RC beams with the same dimensions and external reinforcement used in this study.

The results confirmed the effectiveness of the EB/SRG system and, in particular, improved performance of the IRS/SRG system in terms of ultimate loads and ductility. They showed that with the IRS technique, a considerably greater debonding strain is obtained. To summarize, the two monotonic beams A and B reach failure loads of 83.70 kN and 93.00 kN, with an increase of 19.22% and 31.30% with respect to the control beams, respectively. The corresponding deflection ductility was estimated at 1.93 and 2.73, respectively.

Starting from these experiences, the degradation in terms of flexural capacity and bond strength for the beams subjected to repeated load was evaluated. The beam reinforced with the SS fiber and EB technique of group B (EB_SS_B) showed an increase in the failure load of 9.27% and a ductility factor of 1.93. Therefore, reductions in the failure load and ductility factor were observed compared to the corresponding monotonically tested beam M/EB_SS_B. The beam EB_SS_A exhibited remarkable bond degradation with detachment of the composite strip by intermediate debonding at the end of the 12th cycle (Figure 6c). In fact, both EB_SS beams were characterized by premature and sudden detachment of the external reinforcement. The weak flexural performance can be associated with poor matrix/steel strip impregnation. The cementitious matrix is not able to optimally saturate the space between two consecutive strands. As reported in paragraph 3.3, for both beams, the strengthening composite (steel strip embedded in the two layers of the cementitious matrix) was completely detached. 

Conversely, the IRS technique considerably improves the structural performance in terms of ultimate load and ductility ratio despite the repeated loading. Indeed, the beams strengthened with the SS textile and IRS technique (IRS/SRG) showed good flexural behavior (Figure 6d and Figure 7d). Both beams IRS_SS_A and IRS_SS_B exhibited a good level of failure load (83.60 and 90.00 kN, respectively) and ductility factor (4.33 and 3.93, respectively). The experimental tests have highlighted that no premature debonding occurred during the load cycles. However, a very limited reduction in the failure load was observed compared to the two monotonically loaded beams (with reference to control beams equal to 19.08% and 27.06%), although the ductility was significantly increased.

On the other hand, the SRG system comprising the GLV steel strip exhibited very good behavior compared to the SS reinforcement. The failure occurred for all beams (EB_GLV_A/B and IRS_GLV_A/B) without debonding, exploiting the entire tensile strength of the composite material, independent of the strengthening technique used. All beams showed similar structural trends to each other as depicted in the load–deflection curves (Figure 6e,f and Figure 7e,f). For these specimens, the yield load of steel bars occurred at 51.70 kN, 56.20 kN, 63.20 kN and 59.80 kN and the steel strands ruptured at 75.70 kN, 75.50 kN, 78.30 kN and 77.70 kN, respectively, with the same percentual increase in the failure load (7.83%, 7.54%, 10.54% and 9.70%, respectively). The midspan ductility at failure was equivalent to 3.17, 3.24, 2.27 and 3.35, respectively. As denoted, the ductility ratios are also rather equal for the four beams. Specifically, it can be concluded that, with the low-density steel strip, the two strengthening techniques EB and IRS produce the same flexural improvement.

In the opinion of the authors, the proper impregnation of the GLV steel strip through the matrix layers allows a homogeneous and strong bond at the concrete/composite interface and an improvement of the interlocking phenomena around the steel strands. The different treatment of the external surface between the two strengthening techniques does not lead to significant changes in the structural behavior. In the case of the SS steel strip, the stress developed at the interface is interrupted by the formation of flexural cracks, and when the repeated loading is active, progressive detachments due to the degradation of the bond strength at the matrix/steel fiber interface occurred. In this regard, the use of matrices with high mechanical properties and structural performance is recommended. An example is represented by geopolymeric matrices. The effective performance of these materials in structural application has been proven/demonstrated in many studies as sustainable alternatives to the traditional epoxy resin and pure cementitious matrix [32,33].

Therefore, it can be concluded that the IRS technique makes the concrete surface much rougher, increasing the effectiveness of the high-density fibers and reducing premature detachment in SRG applications.

On the basis of the obtained results, it is possible to correlate at failure the mechanical properties of the steel strengthening systems with the stress in the composite systems. The quality of the bond of the composite system can be also associated with observed failure modes (detailed in paragraph 3.3). Specifically, the fiber with low mass per unit of area has the same behavior regardless of the strengthening technique with the exploitation of all of the strength. The failure is manifested by the total rupture of the strengthening fiber. On the other hand, the fiber with high mass per unit of area shows greater efficiency when applied with the IRS strengthening technique, which guarantees suitable protection against debonding phenomena. The failure mode involves the formation of a wider crack pattern with a progressive detachment of the external reinforcement.

### 3.2. Midspan Deflection Curves and Recorded Strains

**Figure 8:**
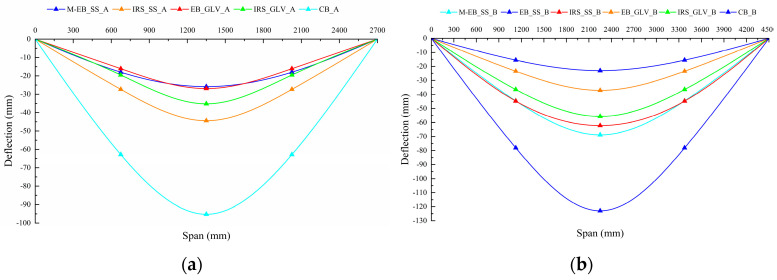
Deflection at failure along effective span: (**a**) group A and (**b**) group B.

The deflections along the effective length of the beams were plotted using the data recorded by the LVDTs arranged at midspan and at quarter span. The application of the external strengthening leads to a reduction in the midspan deflections under maximum load compared with the unstrengthened beams, as shown in Figure 8a,b, respectively. In general, this reduction is directly proportional to the amount of area of the external strengthening. The experimental curves do not clearly highlight this behavior. Evidently, the beams strengthened with SS fibers undergo greater deflections due to the influence of load cycles and the local detachments of the steel strips. However, the IRS technique shows greater deflections than the EB technique, entailing an increase in ductility.

**Figure 9:**
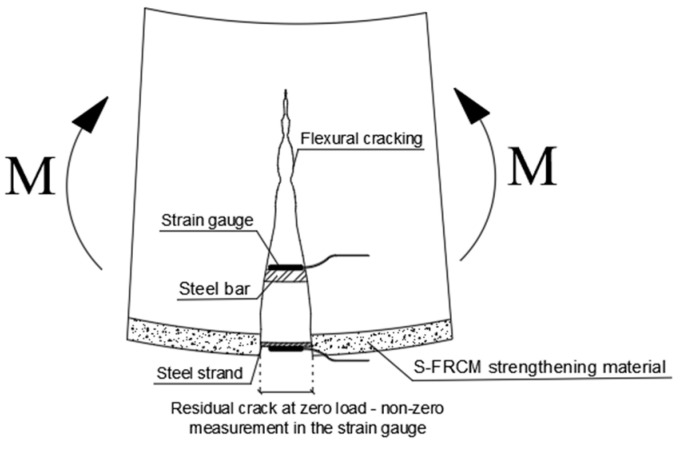
Residual strain phenomena schematization in the tensile zone of the beam.

**Figure 10:**
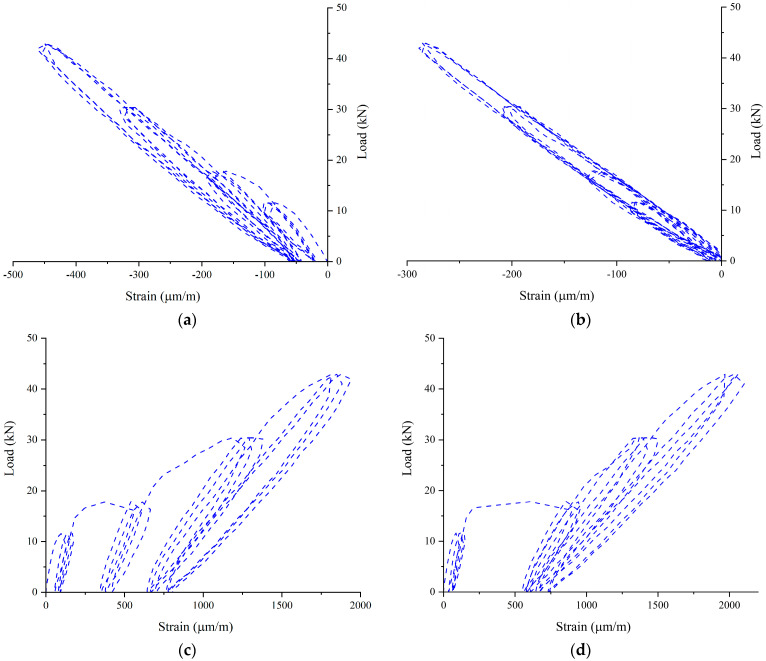
Load–strain curves of EB_SS_B specimen for the first 12 cycles: (**a**) concrete; (**b**) top steel bar; (**c**) bottom steel bar; and (**d**) external fiber.

By focusing the attention on the monitored strains, at the end of each load cycle, nonzero values on internal steel reinforcement bars and the external reinforcement fibers were observed. In the tension zone, after the cracking, the concrete has negligible tension capacity, and the intact concrete between cracks increases the flexural stiffness of the RC beams (tension-stiffening effect). The tensile forces occurring in the crack are absorbed by the internal reinforcement and the external composite. By removing the load, the crack width does not return to its initial position and a part of the residual strain is recorded by the strain gauges. This strain does not represent a plastic accumulation because the cycles are carried out below the yield strength of the steel bars. Conversely, the compressed part of the beam (top concrete surface and top steel bars) shows no residual strain at zero loads. The schematization of the phenomenon is shown in Figure 9, and for the sake of brevity, the load–strain curves of the EB_SS_B beam are shown in Figure 10. The same phenomenon was observed for all tested beams under repeated loading.

**Figure 11:**
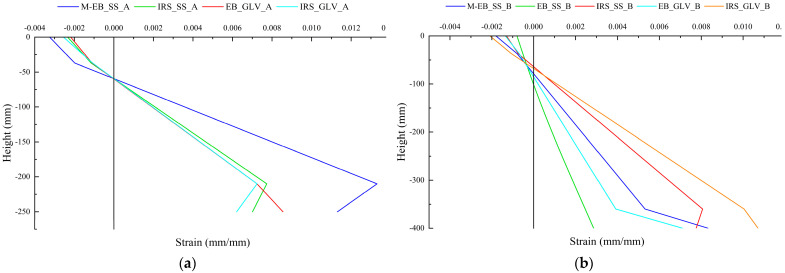
Strain profile at failure: (**a**) group A and (**b**) group B.

The strain profiles at failure are depicted for both groups in Figure 11a,b. The slope of the profile represents the curvature of the sections. The beams strengthened with IRS/SRG systems (IRS_GLV_A, IRS_GLV_B, IRS_SS_A and IRS_SS_B) showed greater local ductility than the EB/SRG systems (EB_GLV_A, EB_GLV_B and EB_SS_B). In detail, the GLV steel strip, thanks to the least amount of external reinforcement, reached the highest strain values than the corresponding SS steel strip. In general, the IRS technique allows beams to lose local and global ductility less (in terms of curvature and displacement).

Finally, the strains at failure load can be read in Table 6 for concrete (ε_c_), top steel bars (ε′_s_), bottom steel bars (ε_s_) and SS/GLV steel fiber (ε_f_). The external steel strip strains are equal to 6.21‰, 8.55‰, 7.01‰ and 11.30‰ for M/EB_SS_A, IRS_SS_A, EB_GLV_A and IRS_GLV_A beams, respectively. The external steel strip strains for M/EB_SS_B, EB_SS_B, IRS_SS_B, EB_GLV_B and IRS_GLV_B beams achieved 8.33‰, 2.86‰, 7.75‰, 7.10‰ and 10.71‰, respectively.

### 3.3. Failure Modes and Crack Patterns

**Figure 12:**
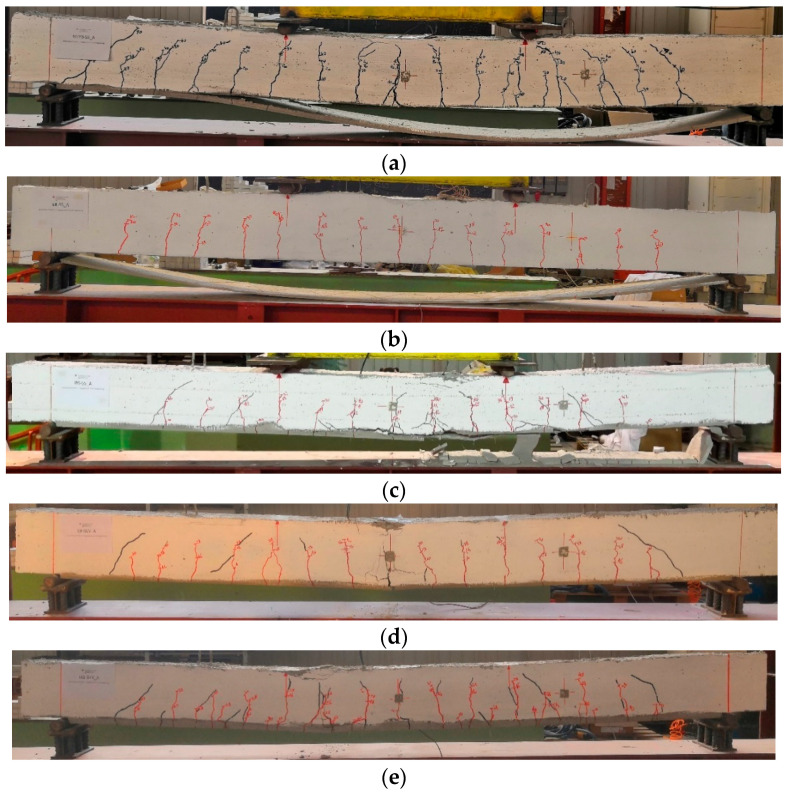
Failure mode and crack patterns of group A: (**a**) M/EB_SS_A; (**b**) EB_SS_A; (**c**) IRS_SS_A; (**d**) EB_GLV_A; and (**e**) IRS_GLV_A.

**Figure 13:**
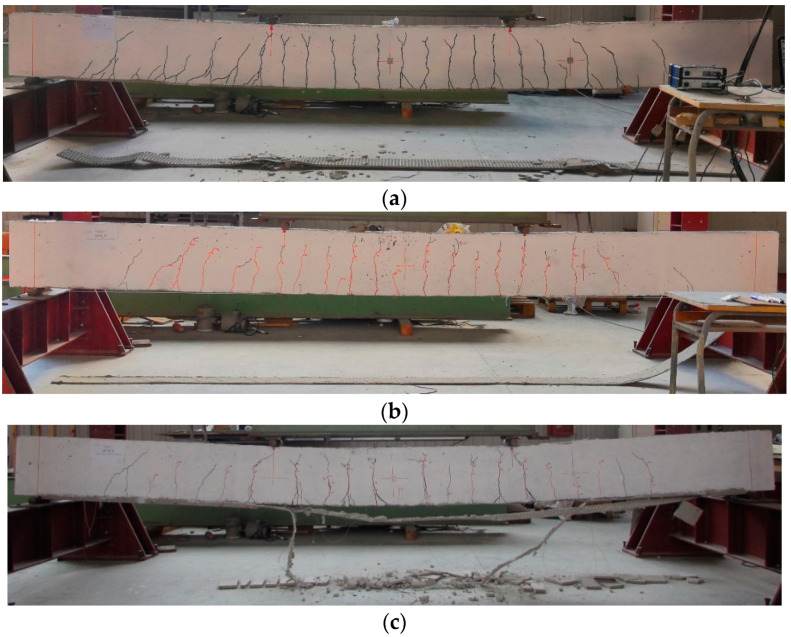

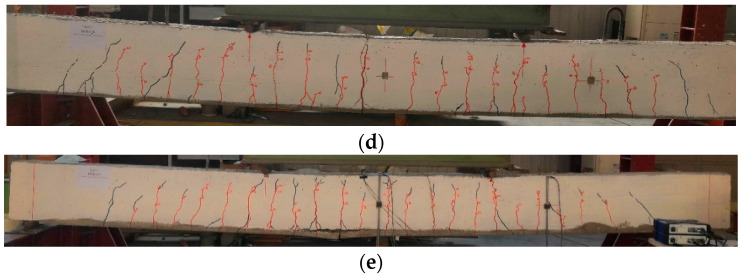
Failure mode and crack patterns of group B: (**a**) M/EB_SS_B; (**b**) EB_SS_B; (**c**) IRS_SS_B; (**d**) EB_GLV_B; and (**e**) IRS_GLV_B.

The crack patterns at failure for each strengthened beam are reported in Figure 12 and Figure 13. The failure modes are represented in the same figures. More precisely, the cracks formed during the load cycles are marked in red, whereas those during the monotonic loading are marked with black. All beams exhibited the classical flexural cracks in the region of constant moment (middle part of the span) with spread and growth in the clear span between the external supports. Near each end region, a few shear cracks developed at high values of the applied load. At the same time, the cracks across the cementitious matrix were observed and marked on the external composite.

The IRS/SRG reinforcement produces a more marked and distributed crack pattern in the lower part of the beam (microcracks at the SRG/concrete interface) compared to beams strengthened with the EB/SRG system. Furthermore, the cracks produced by monotonic loading are more visible, indicating a stronger bond after the repeated loading phase. The number of cracks increases in the beams strengthened with the GLV steel strip compared to the other ones. Finally, in the monotonically tested beams, a greater height of the neutral axis is highlighted, as can be seen from the figures.

For all beams strengthened with the stainless steel strip (SS), the failure mechanisms were controlled by intermediate debonding (Figure 12a,b and Figure 13a,b). 

Specifically, the total delamination of the composite strip occurred in the externally bonded (EB) strengthened beams. It was related to the complexity, observed during the strengthening phase, to achieve proper impregnation between the stainless steel strip and the matrix. This drawback is partially overcome by using the IRS technique. In fact, the IRS-strengthened beams showed partial debonding in the middle section with detachment of part of the lower matrix layer, providing ductility behavior during the failure response, with damage developed through the matrix to steel strip interface. The specimen IRS_SS_A failed by concrete crushing with minimal debonding of the strengthening system (Figure 12c).

**Figure 14:**
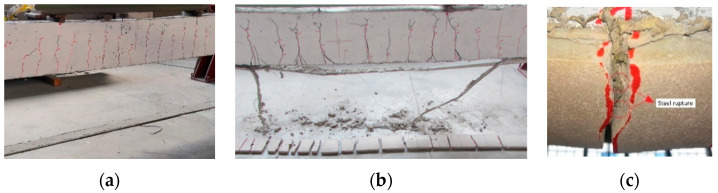
Detail of the failure mode observed: (**a**) complete intermediate debonding of EB_SS beams; (**b**) partial intermediate debonding of IRS_SS beams; and (**c**) rupture of the external steel strip of EB/IRS_GLV beams.

As mentioned in subparagraph 3.1, all beams strengthened with low-density steel fibers (GLV) exploited the steel strand tensile rupture of the composite system with a wide flexural crack in the central span, as depicted in Figure 12d,e and Figure 13d,e. This type of failure is independent of the strengthening technique used. The crushing of the concrete occurred in the beams of group A (EB_GLV_A and IRS_GLV_A) (Figure 12d,e). Figure 14 reports a detailed summary of the main failure modes that occurred.

## 4. Conclusions

The present study aims to provide the first assessment of the influence of pseudocyclic loads on the behavior of SRG systems in the flexural strengthening of RC beams.

A series of repeated loading tests on full-scale reinforced concrete (RC) beams strengthened with steel-reinforced grout (SRG) systems has been performed in order to investigate the structural behavior and the possible effect on the debonding phenomena. The influence of a few cycles at elevated loads (up to 80% of the yield load of tension steel bars) suggests high bond degradation in some specimens. Finally, the strengthening techniques (EB and IRS) were compared to show their feasibility and effectiveness. The findings of this study can be summarized in some points:

- The results of the experimental tests highlighted greater effectiveness of the SRG system with low mass per unit of area (GLV steel strip) in the response of the specimens under repeated loading. The high penetration capacity of the cementitious matrix produces a strong and improved bond. The quality of the bond leads to the failure mode. Both strengthening techniques (EB and IRS) provide similar structural behavior.
- Using a high mass per unit of steel fiber area (SS steel strip), to prevent any damage in the strengthening composite, great attention is required in restoring the RC beams before applying the external strengthening system. In this case, the IRS technique has been confirmed to be a significantly more effective technique in strengthening, also showing greater ductility than the EB technique. The IRS technique allows a better fibers/matrix bond.
- The beams strengthened with the EB/SRG system showed higher flexural capacity than the unstrengthened beams under monotonic loading. On the other hand, the experimental results point out the severe bond degradation of the beams strengthened with stainless steel fiber and subjected to repeated loading.
- The IRS/SRG system response was more ductile (with a deflection ratio that ranges from 3.17 to 4.33) and more effective in terms of failure load (percentual increments range between 19.08% and 27.06%) and exploitation of all materials.
- In general, for all strengthened specimens, an increase in load capacity and a decrease in ductility in terms of displacements and curvatures were found compared to the unstrengthened specimens.
- The ductility index, defined as the ratio between the ultimate displacement and the displacement at the end of the elastic phase, is almost equal in the GLV-strengthened beams and in all specimens strengthened using the IRS technique.

In order to expand knowledge, the results obtained in this study need to be more deeply investigated and confirmed with more consistent experimental programs, considering both cyclic and fatigue behavior. Finally, the issue concerning the effectiveness of external anchor devices (U-shaped anchors, nail anchors, and so on) could be clarified.

## Figures and Tables

**Table 1:** Geometrical configuration of the RC beams.

Group ID	L (mm)	l (mm)	h (mm)	d (mm)	l/h	a (mm)	a/d
A	3000	2700	250	218	12	900	4.1
B	4800	4500	400	368		1500	

**Table 2:** Material properties of concrete and internal steel bars.

		f_cm_ (MPa)	f_ctm_ (MPa)	f_ym_ (MPa)	f_tm_ (MPa)
Concrete	28 days	33.45 (0.108)	3.76 (0.119)	-	-
	day of tests	34.81 (0.095)	2.54 (0.107)	-	-
Internal steel bars	Φ8	-	-	510.57 (0.013)	595.57 (0.006)
	Φ14	-	-	501.20 (0.005)	606.60 (0.005)

**Table 3:** Geometrical properties of the steel strips.

Steel Fiber	A_f,str_ (mm^2^)	s (cords/cm)	ω_f_ (g/m^2^)	t_f_ (mm)
Stainless steel (SS)	0.470	1.57	2200	0.235
Galvanized steel (GLV)	0.519	5.20	650	0.083

**Table 4:** Mechanical properties of the cementitious matrix.

f_cmm_ (MPa)	f_tmm_ (MPa)	E_m_ (MPa)
43.67	5.77	13,607.84

**Table 5:** Test results.

Trave	ρ_f_	F_cr_ (kN)	F_sy_ (kN)	F_u_ (kN)	δ_cr_ (mm)	δ_sy_ (mm)	δ_u_ (mm)	Δ_F_	μ_δ_	Δ_μ_	Failure Mode
CB_A	-	16.71	68.38	70.20	1.22	14.30	98.11	-	6.86	-	CC
M/EB_SS_A	0.00076	13.40	70.20	83.70	0.99	13.38	25.85	1.19	1.93	0.28	ID
IRS_SS_A	0.00076	-	56.70	83.60	-	10.24	44.37	1.19	4.33	0.63	ID
EB_GLV_A	0.00026	-	51.70	75.70	-	8.49	26.89	1.08	3.17	0.46	SR
IRS_GLV_A	0.00026	-	56.20	75.50	-	10.85	35.21	1.08	3.24	0.47	SR
CB_B	-	24.95	67.18	70.83	1.91	19.62	123.75	-	6.31	-	CC
M/EB_SS_B	0.00067	16.80	77.20	93.00	1.94	25.22	68.76	1.31	2.73	0.43	ID
EB_SS_B	0.00067	-	50.70	77.40	-	11.92	22.98	1.09	1.93	0.31	ID
IRS_SS_B	0.00067	-	64.10	90.00	-	15.83	62.18	1.27	3.93	0.62	ID
EB_GLV_B	0.00023	-	63.20	78.30	-	16.35	37.10	1.11	2.27	0.36	SR
IRS_GLV_B	0.00023	-	59.80	77.70	-	16.63	55.68	1.10	3.35	0.53	SR

Legend: CC = concrete crushing; ID = intermediate debonding; SR = steel strand rupture.

**Table 6:** Strains at failure.

Beam ID	ε_c_	ε′_s_	ε_s_	ε_f_
M/EB_SS_A	0.00255	0.00108	0.00724	0.00621
IRS_SS_A	0.00218	0.00110	0.00724	0.00855
EB_GLV_A	0.00234	0.00115	0.00773	0.00701
IRS_GLV_A	0.00324	0.00198	0.0133	0.0113
M/EB_SS_B	0.00181	0.000791	0.00531	0.00833
EB_SS_B	0.000798	0.000520	0.00246	0.00286
IRS_SS_B	0.00130	0.000740	0.00806	0.00775
EB_GLV_B	0.00135	0.00069	0.00392	0.00710
IRS_GLV_B	0.00209	0.00106	0.0100	0.0107

## Data Availability

Data sharing not applicable.
